# Elevated cardiovascular risk among adults with obstructive and restrictive airway functioning in the United States: a cross-sectional study of the National Health and Nutrition Examination Survey from 2007–2010

**DOI:** 10.1186/1465-9921-13-115

**Published:** 2012-12-13

**Authors:** Earl S Ford, Anne G Wheaton, David M Mannino, Letitia Presley-Cantrell, Chaoyang Li, Janet B Croft

**Affiliations:** 1 Division of Population Health, National Center for Chronic Disease Prevention and Health Promotion, Centers for Disease Control and Prevention, Atlanta, GA, USA; 2 Department of Preventive Medicine and Environmental Health, University of Kentucky College of Public Health, Lexington, KY, USA; 3 Division of Behavioral Surveillance, Public Health Surveillance Program Office, Office of Surveillance, Epidemiology, and Laboratory Services, Centers for Disease Control and Prevention, Atlanta, GA, USA; 4 Centers for Disease Control and Prevention, 4770 Buford Highway, MS K67, Atlanta, GA, 30341, USA

**Keywords:** Chronic obstructive pulmonary disease, Cardiovascular diseases, Risk factors, Spirometry

## Abstract

**Background:**

Reasons for the excess risk for cardiovascular disease among people with chronic obstructive pulmonary disease remain unclear. Our objective was to examine the cardiovascular risk profile for adults with obstructive and restrictive impairments of lung functioning in a representative sample of adults from the United States.

**Methods:**

We used data from adults aged 20–79 years who participated in the National Health and Nutrition Examination Survey from 2007 to 2010 and had a pulmonary function test. The severity of obstructive impairment was defined by adapting the Global Initiative for Chronic Obstructive Lung Disease criteria.

**Results:**

Among 7249 participants, 80.9% had a normal pulmonary function test, 5.7% had a restrictive impairment, 7.9% had mild obstructive impairment, and 5.5% had moderate or severe/very severe obstructive impairment. Participants with obstructive impairment had high rates of smoking and increased serum concentrations of cotinine. Compared to participants with normal pulmonary functioning, participants with at least moderate obstructive impairment had elevated concentrations of C-reactive protein but lower concentrations of total cholesterol and non-high-density lipoprotein cholesterol. Among participants aged 50–74 years, participants with at least a moderate obstructive impairment or a restrictive impairment had an elevated predicted 10-year risk for cardiovascular disease.

**Conclusions:**

The high rates of smoking among adults with impaired pulmonary functioning, particularly those with obstructive impairment, point to a need for aggressive efforts to promote smoking cessation in these adults. In addition, adults with restrictive impairment may require increased attention to and fine-tuning of their cardiovascular risk profile.

## Background

Over 137,000 deaths were preliminarily ascribed to chronic lower respiratory diseases in 2010 resulting in this group of lung disorders continuing to be ranked for the third straight year as the third leading cause of death in the United States [1]. Over 95% of these respiratory deaths are attributed to chronic obstructive pulmonary disease (COPD), which includes emphysema and chronic bronchitis. People with COPD have a higher mortality rate than those who do not have COPD [2]. Furthermore, people with COPD are at increased risk of developing or dying from cardiovascular disease [2-10]. Also, impaired lung function as measured by the forced expiratory volume in one second (FEV1) flow is inversely associated with cardiovascular events [11,12]. These observations raise questions about the cardiovascular risk profile among people with COPD. Among deaths from bronchitis, emphysema, and chronic airway obstruction in the United States in 2000–2004, 82.2% were attributed to cigarette smoking [13]. Because smoking is considered the major cause of COPD in many societies, the high prevalence of smoking among people with COPD likely accounts for part or perhaps most of the increased risk. However, the possible contribution to increased mortality from cardiovascular disease by other risk factors is less well understood. Clarification of the possible cardiovascular factors that may increase the risk for cardiovascular disease mortality would help in directing the clinical management of adults with COPD in order to mitigate this extra risk. Therefore, the primary objective of the present study was to compare cardiovascular risk factors between adults with and without COPD determined from spirometry. In addition, we also examined the cardiovascular risk profile among adults with a restrictive impairment.

## Methods

Participants of the National Health and Nutrition Examination Survey (NHANES) 2007–2010 were selected by using a stratified multistage probability sample. After agreeing to participate, invitees were interviewed in their homes and asked to attend an examination in the mobile examination center. Attendees completed additional questionnaires, underwent various examinations, and were phlebotomized. Response rates for the interview and examination were 78% and 75%, respectively, in NHANES 2007–2008, and 79% and 77%, respectively, in NHANES 2009–2010. The NHANES website offers detailed information about the surveys [14]. NHANES 2007–2010 was approved by the National Center for Health Statistics (NCHS) Research Ethics Review Board.

Spirometry was offered to participants aged 6–79 years in NHANES 2007–2010 [15,16]. Participants were excluded for the following reasons: current chest pain; physical problem with forceful expiration; use of supplemental oxygen; recent surgery of the eye, chest or the abdomen; recent heart attack, stroke, tuberculosis exposure, or coughing up of blood; and history of detached retina or a collapsed lung. Participants were asked to provide three acceptable maneuvers using Ohio 822/827 dry-rolling seal volume spirometers [16].

We used predictive equations for forced expiratory volume in 1 second (FEV1) and forced vital capacity (FVC) that were derived from NHANES III data [17]. We applied the Global Initiative for Chronic Obstructive Lung Disease (GOLD) classification, which was designed for use with postbronchodilator results, to the prebronchodilator spirometric results and established the following categories of obstructive impairment: severe obstructive impairment (FEV1/ FVC <0.70 and FEV1 <50% predicted), moderate obstructive impairment (FEV1/FVC <0.70 and FEV1 50 to <80% predicted), and mild obstructive impairment (FEV1/FVC <0.70 and FEV1 ≥80% predicted). Because the number of participants with severe obstructive impairment was limited, we combined participants with moderate and severe obstructive impairment into one group. Participants with a FEV1/FVC ratio ≥0.70 were considered as not having obstructive impairment [18]. In addition, we also defined a category of restrictive impairment (FEV1/FVC ≥0.70 and FVC <80% predicted) for some analyses.

Participants who had an obstructive impairment as defined by FEV1/FVC less than 70% or the lower limit of normal were eligible for bronchodilator testing. Exclusion criteria included the following: cardiovascular conditions (uncontrolled blood pressure, irregular pulse on examination, taking medication for major arrhythmia, implanted defibrillator, or history of congenital heart disease), taking certain prescription medications (monamine oxidase inhibitors, anticonvulsants, tricyclic antidepressants, current treatment for cardiac disease, potassium lowering drugs), recent use of β2-adrenergic bronchodilators, previous adverse reaction to albuterol, pregnancy, and breastfeeding. In all, almost half of adults did not complete this portion of the protocol because of exclusion criteria or other reasons. We classified the postbronchodilator results as above to create groups with mild, moderate, and severe or very severe obstructive impairment and retained the groups with normal lung function and restrictive impairment based on the prebronchodilator results.

We examined the following cardiovascular risk factors: smoking status, concentration of cotinine, hypertension, hypercholesterolemia, low concentration of high-density lipoprotein cholesterol (<1.03 mmol/L in men and <1.29 mmol/L in women), non-high-density lipoprotein cholesterol ≥3.36 mmol/L, low-density lipoprotein cholesterol ≥2.59 mmol/L, C-reactive protein >3 mg/dl, diagnosed diabetes, body mass index ≥30 kg/m^2^, abdominal obesity (≥102 cm in men, ≥88 cm in women), leisure-time physical activity <150 minutes per week. A current smoker was defined as someone who had smoked at least 100 cigarettes during his or her lifetime and reported still currently smoking. A former smoker was defined as someone who had smoked at least 100 cigarettes during his or her lifetime but reported having stopped smoking. A never smoker was defined as someone who had not smoked at least 100 cigarettes during his or her lifetime. Up to four attempts were made to measure blood pressure. The average of the last two measurements of blood pressure for participants who had three measurements, the last measurement for participants with only two measurements, and the only measurement for participants who had one measurement were used. Hypertension was defined as a systolic blood pressure ≥140 mm Hg or a diastolic blood pressure ≥90 mm Hg or the self-reported use of antihypertensive medications. Body mass index (kg/m^2^) was calculated from measured weight and height. Waist circumference at the level of the ilium was measured to nearest 1 mm at the end of normal expiration. If participants responded affirmatively to the question “Have you ever been told by a doctor or health professional that you have diabetes or sugar diabetes?”, they were considered to have diagnosed diabetes. Participants who reported having borderline diabetes were considered as not having diagnosed diabetes.

Serum concentrations of cotinine were measured with isotope-dilution high-performance liquid chromatography /atmospheric pressure chemical ionization tandem mass spectrometric method. Concentrations of total cholesterol were measured with an enzymatic method on a Roche Modular P chemistry analyzer (Roche Diagnostics, Indianapolis, IN) during 2007–2008 and on a Beckman Coulter UniCel DxC800 Synchron Clinical System (Beckman Coulter, Inc., Brea, CA) during 2009–2010. Concentrations of high-density lipoprotein cholesterol were measured after precipitation with magnesium/dextran sulfate on a Roche Modular P chemistry analyzer (Roche Diagnostics, Indianapolis, IN). Concentrations of non-high-density lipoprotein cholesterol were calculated by subtracting concentrations of high-density lipoprotein cholesterol from those of total cholesterol. Concentrations of low-density lipoprotein cholesterol were calculated by using the Friedewald equation only for fasting participants who had fasted from 8.5 to 24 hours and attended the morning examination. Concentrations of C-reactive protein were measured by latex-enhanced; nephelometry on a Dade Behring Nephelometer II Analyzer System (BNII) (Dade Behring Diagnostics Inc., Somerville, NJ).

We used a multivariable risk algorithm derived from Framingham data to calculate the predicted 10-year cardiovascular risk for adults aged 30–74 years [19]. This algorithm incorporates age, concentrations of total cholesterol and high-density lipoprotein cholesterol, systolic blood pressure, hypertension treatment status, smoking, and diabetes. In this algorithm, diabetes is defined on the basis of glucose measurements. For the purposes of calculating cardiovascular risk, diabetes was defined as having a concentration of fasting plasma glucose ≥7.0 mmol/L or the self-reported use of insulin or oral hypoglycemic medications. Because fasting plasma glucose was measured only for a subsample of participants who attended the morning examination, analyses of predicted 10-year cardiovascular risk were limited to fasting participants who attended the morning examination.

In addition, we included the following sociodemographic covariates: age, gender, self-reported race or ethnicity (white, African American, Mexican American, and other), and educational level (less than 12 years, high school graduate or equivalent, education beyond high school) . We also included self-reported congestive heart failure (Has a doctor or other health professional ever told you that you had congestive heart failure?).

Most of the analyses were limited to participants aged 20–79 years who had a spirometric examination in the mobile examination center. Age-adjustment with three age groups (20–39 years, 40–59 years, and 60–79 years) was performed by using the direct method and the projected year 2000 U.S. population. Differences in means and proportions were tested with t-tests. Prevalence ratios that were generated from log-binomial regression analyses were used to examine associations between cardiovascular risk factors and respiratory impairment independent of sociodemographic factors and other cardiovascular risk factors. We used SAS and SUDAAN, the latter to account for the complex sampling design of the surveys. Sample sizes shown in the text and tables are unweighted numbers. Sampling weights were used to produce estimates (means, percentages, and prevalence ratios) that are representative of the civilian noninstitutionalized population in the United States.

## Results

Of the 10981 participants aged 20–79 years who had an examination in NHANES 2007–2010, 9172 had a spirometric examination, and 9047 had values for FEV1 and FVC. Excluding participants who lacked a value for height reduced the number of participants to 9024. Limiting participants to those who had a complete examination and met or exceeded American Thoracic Society data collection standards left 7890 participants. After additional exclusions for participants with missing data for other study variables, 7249 participants were included in the analysis based on prebronchodilator testing and 6816 based on postbronchodilator testing.

Among the 7249 participants, 80.9% had a normal pulmonary function test, 5.7% had a restrictive impairment, 7.9% had a mild obstructive impairment, and 5.5% had a moderate or severe obstructive impairment. Participants with a normal pulmonary function test, restrictive impairment, and obstructive impairment differed in mean age, gender distribution, race or ethnic distribution, and educational distribution (Table 1).

**Table 1:** Age-adjusted means and percentages (standard error) of sociodemographic and cardiovascular factors among 7249 U.S. adults aged 20–79 years, by categories of respiratory impairment based on prebronchodilator data, National Health and Nutrition Examination Survey 2007-2010

	**Respiratory impairment**						
			**Obstructive impairment**				
	**None (N=5792)**	**Restrictive (N=478)**	**Mild (N=551)**	**Moderate/severe/very severe (N=428)**	**P restrictive‡**	**P mild OI‡**	**P moderate/ severe OI‡**
Age (years)	42.5 (0.3)	50.1 (0.9)	54.9 (0.7)	55.2 (0.7)	<0.001	<0.001	<0.001
Men (%)	46.5 (0.7)	56.4 (3.1)	66.0 (3.0)	58.4 (3.6)	0.002	<0.001	0.004
Race or ethnicity (%)							
White	70.4 (2.3)	54.7 (5.1)	84.1 (2.8)	78.0 (4.4)	0.001	<0.001	0.042
African American	9.9 (1.0)	9.3 (1.4)	7.9 (1.9)	7.7 (1.6)			
Mexican American	9.3 (1.4)	8.8 (1.9)	3.4 (1.1)	1.2 (0.4)			
Other	10.4 (1.1)	27.2 (4.0)	4.5 (1.3)	13.1 (3.6)			
Education (%)							
<High school	16.4 (0.9)	19.7 (2.0)	17.2 (2.5)	22.5 (3.4)			
High school graduate or equivalent	22.2 (0.9)	25.0 (3.8)	28.4 (3.5)	34.4 (3.8)			
>High school (%)	61.4 (1.4)	55.3 (4.1)	54.4 (4.0)	43.1 (4.9)	0.112	0.063	<0.001
Smoking status (%)							
Current	18.8 (0.7)	24.2 (3.2)	35.7 (2.4)	48.6 (3.8)	0.081	<0.001	<0.001
Former	23.0 (0.9)	23.2 (2.8)	26.6 (3.0)	26.4 (3.7)			
Never	58.2 (1.2)	52.6 (4.3)	37.8 (3.6)	25.0 (3.4)			
Cotinine >10 ng/ml (%)	22.9 (0.8)	29.3 (3.5)	44.5 (3.5)	53.1 (4.3)	0.048	<0.001	<0.001
Hypertension (%)	25.1 (0.8)	34.1 (2.9)	25.8 (2.4)	30.8 (3.5)	0.003	0.761	0.101
Total cholesterol ≥5.17 mmol/L (%)	45.2 (0.9)	41.4 (2.6)	43.4 (3.6)	36.8 (3.4)	0.212	0.619	0.027
High-density lipoprotein cholesterol <1.03 mmol/L in men, <1.29 mmol/L in women (%)	32.1 (1.1)	50.2 (2.9)	26.7 (3.2)	36.4 (5.1)	<0.001	0.085	0.397
Non-high-density lipoprotein cholesterol ≥130 mg/dl (%)	62.0 (0.8)	61.8 (2.9)	62.2 (3.6)	53.9 (3.9)	0.946	0.951	0.048
Low-density lipoprotein cholesterol ≥2.59 mmol/L* (%)	67.7 (0.9)	69.0 (4.0)	72.4 (4.9)	61.1 (6.0)	0.747	0.350	0.290
C-reactive protein >3 mg/l (%)	30.1 (0.7)	48.8 (3.1)	22.9 (2.5)	34.1 (4.6)	<0.001	0.010	0.371
FPG ≥7.0 mmol/L or diagnosed diabetes† (%)	9.1 (0.7)	25.8 (3.7)	3.2 (0.7)	10.0 (1.8)	<0.001	<0.001	0.678
Diagnosed diabetes (%)	6.1 (0.5)	18.6 (2.3)	3.9 (0.6)	7.3 (1.3)	<0.001	0.002	0.414
Body mass index ≥30 kg/m2 (%)	35.4 (1.0)	51.4 (2.9)	21.6 (2.7)	30.0 (4.0)	<0.001	<0.001	0.206
Abdominal obesity (%)	53.7 (1.1)	65.1 (2.9)	35.8 (2.9)	50.2 (4.0)	<0.001	<0.001	0.434
Leisure-time physical activity <150 min/week (%)	59.9 (1.5)	69.9 (2.8)	55.3 (3.0)	65.3 (4.3)	0.004	0.210	0.225
Congestive heart failure (%)	0.8 (0.1)	3.2 (0.8)	0.9 (0.2)	—§	0.003	0.550	0.069
10-Year cardiovascular risk†							
<10%	77.3 (0.9)	68.9 (3.9)	73.2 (2.3)	68.9 (2.7)			
10-20%	14.6 (1.0)	16.4 (2.8)	15.4 (2.2)	14.4 (2.6)			
>20%	8.1 (0.5)	14.7 (2.3)	11.4 (1.2)	16.7 (2.0)	0.004	0.012	<0.001

*OI* obstructive impairment.

*Fasting subsample. Sample sizes are 2537, 220, 270, and 180, respectively.

†Fasting subsample. Sample sizes are 2581, 227, 272, and 183, respectively.

‡P-values are for comparison with participants with no respiratory impairment.

§Relative standard error >30%.

**Figure 1:**
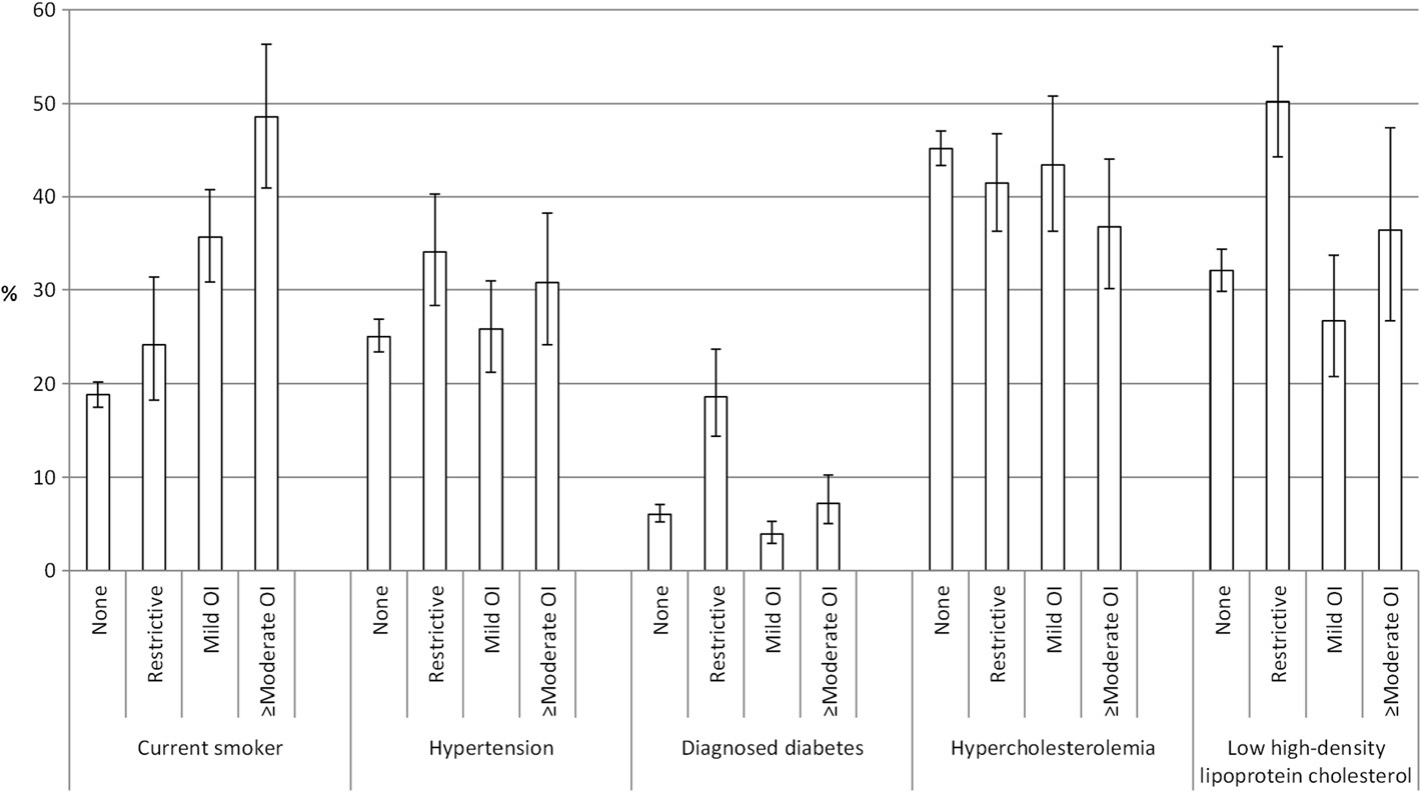
**Age-adjusted percentages (standard error) of selected cardiovascular factors among 7249 U.S. adults aged 20-79 years, by categories of respiratory impairment based on prebronchodilator data, National Health and Nutrition Examination Survey 2007-2010.** OI = obstructive impairment.

After age-adjustment, respiratory function status was significantly associated with all cardiovascular risk factors except low-density lipoprotein cholesterol (Table 1, Figure 1). However, the pattern of risk differed between adults with a restrictive impairment and an obstructive impairment. Compared to adults with normal pulmonary function, those with a restrictive impairment were more likely to have an elevated concentration of cotinine, hypertension, low levels of high-density lipoprotein cholesterol, an increased concentration of C-reactive protein >3 mg/l, an increased prevalence of diabetes, obesity, and abdominal obesity, an increased level of physical activity, and an elevated 10-year cardiovascular risk of >20%. Compared to adults with normal pulmonary function, those with a mild obstructive impairment were more likely to be a current smoker, have an elevated concentration of cotinine, have a decreased concentration of C-reactive protein, have a lower prevalence of diabetes, obesity, and abdominal obesity, and have an elevated 10-year cardiovascular risk of >20%. Compared to adults with normal pulmonary function, those with a moderate or severe obstructive impairment were more likely to be a current smoker and have an elevated concentration of cotinine, whereas they were less likely to have an elevated concentration of total cholesterol and non-high-density lipoprotein cholesterol. However, persons with moderate or severe obstructive impairment had a higher age-adjusted percentage with an overall 10-year cardiovascular risk >20% than persons with normal pulmonary function (16.7% vs. 8.1%, p <0.001).

The results from multivariate analyses using prebronchodilator data are shown in Table 2. Compared to participants with a normal pulmonary function test, those with a restrictive impairment were more likely to be a current smoker, to have an elevated concentration of cotinine, to have low levels of high-density lipoprotein cholesterol, to have an elevated concentration of C-reactive protein >3 mg/l, and to have an elevated prevalence of diagnosed diabetes and obesity. Compared to adults with normal pulmonary function, those with a mild obstructive impairment were more likely to be a current smoker, have an elevated concentration of cotinine, and have a lower prevalence of diabetes, obesity, and abdominal obesity. Compared to adults with normal pulmonary function, those with at least moderate obstructive impairment were more likely to be a current smoker and have an elevated concentration of cotinine, were less likely to have an elevated concentration of total cholesterol and non-high-density lipoprotein cholesterol, and were more likely to have an elevated concentration of C-reactive protein.

**Table 2:** Adjusted* prevalence ratios (95% **confidence interval) for cardiovascular risk factors among 7249 U.S. adults aged 20–79 years, by categories of respiratory impairment based on prebronchodilator data, National Health and Nutrition Examination Survey 2007-2010**

	**Respiratory impairment**				
			**Obstructive impairment**		
	**None**	**Restrictive**	**Mild**	**Moderate/severe/ very severe**	**P†**
Current smoking	1.00	1.23 (1.01, 1.51)	1.68 (1.40, 2.00)	2.26 (1.90, 2.70)	<0.001
Cotinine >10 ng/ml	1.00	1.23 (1.04, 1.46)	1.62 (1.37, 1.90)	2.00 (1.69, 2.36)	<0.001
Hypertension	1.00	0.99 (0.86, 1.15)	1.00 (0.88, 1.15)	1.03 (0.88, 1.21)	0.974
Total cholesterol ≥5.17 mmol/L	1.00	0.91 (0.78, 1.06)	0.90 (0.80, 1.02)	0.85 (0.73, 0.99)	0.068
High-density lipoprotein cholesterol <40 mg in men, <1.29 mmol/L in women	1.00	1.18 (1.04, 1.34)	0.95 (0.78, 1.16)	1.04 (0.85, 1.26)	0.188
Non-high-density lipoprotein cholesterol ≥130 mg/dl	1.00	0.92 (0.83, 1.01)	0.93 (0.84, 1.02)	0.86 (0.78, 0.95)	0.017
Low-density lipoprotein cholesterol ≥2.59 mmol/L‡	1.00	0.95 (0.84, 1.08)	1.00 (0.89, 1.13)	0.91 (0.80, 1.04)	0.514
C-reactive protein >3 mg/l	1.00	1.21 (1.05, 1.40)	1.02 (0.89, 1.18)	1.17 (1.03, 1.33)	0.014
Diagnosed diabetes	1.00	1.84 (1.40, 2.41)	0.70 (0.50, 0.99)	1.01 (0.68, 1.51)	<0.001
Diagnosed diabetes of fasting plasma glucose ≥7.0 mmol/L§	1.00	1.65 (1.19, 2.30)	0.45 (0.30, 0.68)	0.83 (0.54, 1.27)	<0.001
Body mass index ≥30 kg/m^2^	1.00	1.16 (1.03, 1.30)	0.60 (0.50, 0.72)	0.83 (0.68, 1.01)	<0.001
Abdominal obesity	1.00	1.07 (0.99, 1.16)	0.75 (0.69, 0.83)	0.96 (0.86, 1.07)	<0.001
Leisure-time physical activity <150 min/week	1.00	1.05 (0.97, 1.14)	0.99 (0.89, 1.10)	1.05 (0.97, 1.13)	0.479

*Adjusted for age, gender, race or ethnicity, educational status, smoking status (except for cotinine concentration), cotinine concentration (except smoking status), systolic blood pressure (except hypertension), high-density lipoprotein cholesterol concentration (except non-high-density lipoprotein cholesterol concentration), non-high-density lipoprotein cholesterol concentration (except high-density lipoprotein cholesterol), C-reactive protein concentration, diagnosed diabetes, body mass index (except waist circumference), waist circumference (except body mass index), leisure-time physical activity, and history of congestive heart failure. Hypercholesterolemia was adjusted for all above factors except non-high-density lipoprotein cholesterol. Low-density lipoprotein cholesterol was adjusted for all factors except non-high-density lipoprotein cholesterol.

†P-value for Satterthwaite adjusted F-test.

‡Sample size is 3207.

§Sample size is 3263.

Because age is an important component of the calculation of predicted 10-year cardiovascular risk and because differences in age among the four groups of respiratory status were present, we calculated 10-year cardiovascular risk for participants 50–74 years and again for those aged 55–74 years (Table 3). For the former group, significantly larger percentages of adults with a restrictive impairment or obstructive impairment had a risk >20% than adults without an impairment of lung function. In the 55–74 year-old age group, only adults with a moderate or worse obstructive impairment were significantly more likely to have an elevated risk for cardiovascular disease, although participants with a restrictive impairment had a borderline significant increased risk for cardiovascular disease (p = 0.061).

**Table 3:** **Adjusted prevalence ratios (95**% **confidence interval) for 10-year cardiovascular risk >20**% **among U.S. adults, by categories of respiratory impairment based on prebronchodilator data, National Health and Nutrition Examination Survey 2007-2009**

	**Respiratory impairment**			
			**Obstructive impairment (OI)**	
	**None**	**Restrictive**	**Mild**	**Moderate/severe/very severe**
**Age 50–74 years**				
No. risk >20% / no. at risk*	224 / 902	53 / 123	61 / 169	53 / 117
Median age (years)	57	60	60	60
Unadjusted prevalence (standard error) (%)	16.5 (1.2)	34.4 (5.6)	24.3 (4.1)	38.5 (4.5)
Unadjusted prevalence ratio	1.00	2.08 (1.54, 2.80)	1.47 (1.04, 2.09)	2.33 (1.72, 3.15)
Adjusted prevalence ratio†	1.00	1.64 (1.16, 2.31)	1.56 (1.13, 2.15)	2.19 (1.62, 2.97)
**Age 55–74 years**				
No. risk >20% / no. at risk*	199 / 640	49 / 99	56 / 143	46 / 95
Median age (years)	61	62	61	63
Unadjusted prevalence (standard error) (%)	23.1 (1.7)	41.5 (6.7)	26.7 (4.8)	43.8 (5.4)
Unadjusted prevalence ratio	1.00	1.79 (1.34, 2.41)	1.16 (0.82, 1.63)	1.90 (1.36, 2.63)
Adjusted prevalence ratio†	1.00	1.40 (0.98, 1.99)	1.22 (0.90, 1.64)	1.84 (1.36, 2.50)

*Unweighted numbers.

†Adjusted for race or ethnicity, education, body mass index, C-reactive protein, and leisure-time physical activity.

### Postbronchodilator results

We repeated the results shown in Tables 1, 2, and 3 using the postbronchodilator data. Additional tables corresponding to Tables 1, 2, and 3 are included in Additional file 1.

## Discussion

Our results show that, compared to adults who have a normal pulmonary function, adults who have impaired lung function, whether restrictive or obstructive in nature, have a number of abnormalities in their cardiovascular risk profile that might help to explain their increased risk for cardiovascular mortality. However, the comparisons of cardiovascular risk profiles with persons who had normal pulmonary function differed somewhat between adults with a restrictive impairment and those with an obstructive impairment. Persons with restrictive impairment showed more abnormalities of cardiometabolic factors compared to the normal group whereas the latter had primarily higher rates of smoking along with evidence of systemic inflammation compared to the normal group. Furthermore, the predicted 10-year cardiovascular risk was increased among adults aged 50–74 years with either a restrictive or obstructive impairment.

Relatively few studies, especially population-based studies in the United States, have compared the cardiovascular risk factor profile in people with COPD to that in people without COPD. In a cohort of 91932 members of the Kaiser Permanente Medical Care Program of Northern California, patients with COPD identified from administrative databases were more likely to have diabetes, hypertension, and hyperlipidemia and to be obese than patients without COPD [6]. In the Copenhagen City Heart Study, the GOLD classification system was used to establish the respiratory status of participants [20]. The unadjusted percentages of elevated concentrations of HbA1c and C-reactive protein as well as the mean concentration of total cholesterol, mean systolic blood pressure, mean body mass index, and current smoking were higher in 1036 participants with COPD than in 4854 without COPD. However, many of the differences were likely attributable to the much higher mean age in participants with COPD. An analysis of patients with COPD from the United Kingdom identified from the General Practice Research Database reported that patients with COPD were more likely to be underweight, less likely to be obese, and less likely to have hypertension than patients without COPD [10]. In another study that used spirometric testing to establish the COPD status, hypertensive patients with COPD (N=1622) were more likely to smoke and have a higher mean systolic blood pressure than hypertensive patients without COPD (N=1184), but the prevalence of diabetes in the two groups did not differ significantly [9]. In a Korean study of 4001 participants aged ≥18 years, those with obstructive lung function had a higher unadjusted mean waist circumference, systolic blood pressure, diastolic blood pressure, and higher concentrations of glucose and triglycerides but lower concentration of high-density lipoprotein cholesterol [21]. Thus, the available data do not paint a consistent picture about risk factors for cardiovascular disease among people with COPD. Some of the inconsistency may be attributable to differences in methods used to identify participants with COPD (identification from administrative data bases, use of pulmonary function test to categorize COPD status), sample size of the studies, definition of risk factors (self-reported data, administrative data bases, measured data), or other characteristics of study populations. Our results confirm that advanced age and a high prevalence of smoking are the two factors that contribute substantially to the risk for cardiovascular disease among U.S. adults with COPD.

Little information about predicted cardiovascular risk in function of pulmonary function status is available. In an Italian study of 12933 participants aged ≥35 years, the CUORE risk score was not significantly associated with the ratio of FEV1/FVC [22].

People with an obstructive impairment continued to smoke at high rates. Consequently, smoking cessation efforts are critical to reducing the risk for cardiovascular disease among these people. A large body of research has examined the effectiveness of numerous approaches to smoking cessation such as clinical interventions (including behavioral approaches and medications), system interventions, and quitlines [23]. Despite this large body of research concerning the best approaches to initiate and sustain smoking cessation, the report “Treating Tobacco Use and Dependence: 2008 Update” acknowledged the need for additional research concerning the effectiveness of counseling among people with COPD. In view of the strong link between smoking and COPD and the high rates of smoking among people with COPD, such research is urgently needed. A meta-analysis of randomized controlled trials found that the combination of smoking cessation counseling plus the use of nicotine replacement therapy yielded the best rate of smoking cessation, followed by the combination of smoking cessation counseling plus the use of antidepressant medications, and smoking cessation counseling alone [24]. Among patients with COPD, both intensive counseling and pharmacotherapy yielded superior cost-effectiveness compared to minimal counseling [25].

Previous studies have shown that COPD is associated with increased concentrations of C-reactive protein [26-28]. Our analysis found that concentrations of C-reactive protein were elevated in adults with moderate or severe COPD but not in those with a mild obstructive impairment. Because C-reactive protein is considered to be a risk factor for cardiovascular disease [29], the presence of a systemic inflammatory component as evidenced by elevated concentrations of C-reactive protein in people with COPD may help to explain the increased risk for cardiovascular disease in this population.

In addition to excess levels of smoking and elevated concentrations of C-reactive protein, several additional physiological disruptions may contribute to the increased risk for cardiovascular disease in people with COPD including increased circulating platelet-monocyte aggregates [30], impaired coronary blood flow [31], endothelial dysfunction [32,33], coagulation abnormalities [32], oxidative stress [34], and increased arterial stiffness [35].

The impact of medications used to treat COPD provides another possible explanation for the increased risk for cardiovascular disease [36]. Evidence has been presented that the use of short-acting anticholinergic agents may increase mortality from cardiovascular disease [37]. The data concerning the effect of long-acting anticholinergic agents on adverse cardiovascular events has generated intense discussion [38-43]. Furthermore, a meta-analysis of randomized controlled trials concluded that the use of beta-2-agonists increased cardiovascular events [44]. Studies concerning the possible effect of inhaled corticosteroids on cardiovascular outcomes have yielded conflicting data with observational studies showing a possible benefit from the use of these agents and data from randomized controlled trials showing no such effect [45]. Because patients with COPD are susceptible to acute exacerbations of their disease in part from respiratory infections, some classes of antibiotics used to treat these infections such as macrolides and quinolones could conceivably result in adverse cardiovascular events [46].

Our results suggest that people with a mild obstructive impairment do not have a particularly adverse set of cardiometabolic risk factors compared to people with normal respiratory function. Thus, the clinical approach to cardiovascular risk reduction in this population should be similar as that for the general population [47-52]. Although research concerning management of cardiovascular risk factors specifically in patients with COPD is limited, some data suggest that the use of beta-blockers in patients may reduce cardiovascular morbidity and mortality. In a study of 1205 patients with COPD undergoing vascular surgery, beta-blocker therapy, particularly high-dose therapy, was associated with reduced mortality [53]. In addition, the use of angiotensin-converting enzyme inhibitors and angiotensin receptor blockers have also been shown to reduce cardiovascular events in cohorts of patients with COPD [54]. Interestingly, some treatments for COPD may provide cardiovascular benefits to patients with COPD. For example, pulmonary rehabilitation in patients with COPD was associated with improvements in aortic pulse wave velocity, blood pressure, and concentration of total cholesterol [55].

Our analyses of the prebronchodilator data indicated that the prevalence of hypercholesterolemia was somewhat lower among participants with at least moderate obstructive impairment. The explanation for this finding is unclear, and it could represent a spurious finding. The age-adjusted self-reported use of cholesterol-lowering medications and mean intakes of saturated fat and polyunsaturated fat were similar for participants with normal lung function and those with at least moderate obstructive impairment (data not shown). Results from other studies yield mixed evidence about a reduced prevalence of hypercholesterolemia among adults with COPD. In a large study from Canada, 7.9% of patients with COPD and 10.9% of patients without COPD had hypercholesterolemia, a difference which was significantly different [7]. Mean concentrations of low-density lipoprotein cholesterol were also lower in a population of patients with COPD evaluated for lung transplantation [56]. In contrast in a Danish study, unadjusted mean concentrations of total cholesterol were positively associated with severity of COPD [20]. In an Italian study, unadjusted mean concentrations of total cholesterol and low-density lipoprotein cholesterol did not differ between participants with and without COPD [9]. Also, unadjusted mean concentrations of total cholesterol and low-density lipoprotein cholesterol did not differ significantly between patients with emphysema and those without this condition in another study [57].

In recent years, the potential role of treatment with statins in reducing morbidity and mortality among people with COPD has drawn substantial interest [58-60]. The use of statins to manage hypercholesterolemia should yield similar benefits in people with COPD as in those without this condition. Because statins have been shown to have pleiotropic actions including anti-inflammatory and antioxidant effects [61] and because COPD has a powerful inflammatory component [62], statins could conceivably modulate the inflammation unleashed by COPD. Whether statins will reduce cardiovascular morbidity and mortality in people with COPD to the same extent as in people without COPD or whether there is an added benefit to the use of statins in people with COPD remains a question to be addressed by future randomized controlled trials. Of note is that a recent study showed that the use of statins was associated with interstitial lung disease among smokers [63].

A heterogeneous set of disorders can produce a restrictive pattern on spirometry [64]. These disorders can be broadly grouped into three categories: decreased lung compliance (interstitial lung disease, pneumonia, sarcoidosis, acute respiratory distress syndrome), decreased muscle strength (neuromuscular disease, dysfunction of diaphragm, injury of the phrenic nerve), and extrapulmonary disease (pleural effusion, pleural thickening, obesity, metabolic syndrome, kyphoscoliosis). In turn, some of these conditions such as interstitial lung disease have a lengthy list of etiologies.

Observational studies have shown that people with a restrictive impairment have an increased mortality rate from all-causes as well as diseases of the circulatory system [65]. Yet, the cardiovascular risk profile of people with restrictive impairment has not been well characterized. In an analysis of 4320 U.S. adults aged 25–74, participants with a restrictive impairment were more likely to be current smokers, be obese, have hypertension, and have diabetes than participants without restriction [65]. A study of 121,965 French participants showed that a restrictive ventilator pattern was associated with several factors (lipids, glucose-blood pressure, and abdominal obesity) derived from a factor analysis of cardiometabolic variables [66]. Furthermore, adults with a restrictive impairment also show evidence of inflammation [27]. In our analysis, the presence of a number of cardiometabolic abnormalities (increased body mass index, low high-density lipoprotein cholesterol, increased diabetes, elevated C-reactive protein) among the adults with a restrictive impairment is broadly consistent with the aforementioned studies and suggests that these adults are at increased risk for cardiovascular disease. Although our analyses did not specifically examine the association between metabolic syndrome and restrictive ventilatory pattern, the significant associations between several of the cardiovascular risk factors and restrictive impairment is consistent with previous studies that have reported associations between metabolic syndrome and restrictive impairment [21,66-69]. The general profile of these adults suggests that, as a group, they might benefit from weight management, improved nutrition, and increased physical activity in addition to smoking cessation. Furthermore, pharmacotherapy to address hyperglycemia or raise concentrations of high-density lipoprotein cholesterol may help to lessen the cardiovascular risk in these adults.

The major strength of this study is the large nationally representative study population of U.S. adults with spirometry and cardiovascular measurements. The principal limitation of our study was the very small number of adults with a very severe obstructive impairment. Therefore, we were unable to generate results for this specific group. Even the number of adults with severe obstructive impairment proved to be limited leading us to group moderate, severe, and very severe obstructive impairment together.

Although the results based on postbronchodilator data in large measure were similar to the results based on prebronchodilator data, these postbronchodilator results should be cautiously interpreted given the substantial attrition of attendance with 25% of participants aged 20–79 years excluded for safety reasons and another 21% of participants not having the postbronchodilator examination. The safety exclusions included a number of reasons that excluded a number of participants at increased risk for cardiovascular disease: uncontrolled blood pressure, irregular pulse on examination, taking medication for major arrhythmia, implanted defibrillator, and current treatment for cardiac disease.

Addressing the cardiovascular risk factors among persons with restrictive or obstructive pulmonary function would provide support to the federal interagency Million Hearts Initiative to prevent 1 million heart attacks and strokes over the next 5 years [70,71]. The proposed effective clinical services that could reduce cardiovascular disease risk, morbidity, and mortality in this patient population include aspirin therapy, blood pressure control, cholesterol management, and smoking cessation [71]. This initiative has been expanded to include community-level policies and programs designed to reduce exposure to tobacco use, policies to reduce sodium content of food, and policies to eliminate artificial trans fatty acids from the diet [70].

## Conclusion

The high prevalence of smoking among adults with an obstructive impairment was the principal abnormal cardiovascular risk factor, whereas a broader set of cardiometabolic abnormalities among adults with a restrictive impairment contributes to the risk for cardiovascular disease. Although both groups with impaired lung functioning would benefit from smoking cessation, many adults with a restrictive impairment require management of other cardiometabolic factors as well. By providing insights about the status of cardiovascular risk factors that may be partially responsible for the excess morbidity and mortality from cardiovascular disease among adults with obstructive and restrictive lung disease, our results may help to increase the understanding of how best to manage cardiovascular risk in these adults.

## Abbreviations

COPD: Chronic obstructive pulmonary disease; FEV1: Forced expiratory volume in one second; FVC: Forced vital capacity; GOLD: Global Initiative for Chronic Obstructive Lung Disease; NHANES: National Health and Nutrition Examination Survey.

## Competing interests

The authors declare that they have no competing interests.

## Authors’ contributions

EF was responsible for the concept and design of the study and took the lead analyzing the data and in drafting the manuscript. AW, DMM, and CL provided critical input in data analysis and interpretation and provided critical revision of the manuscript for important intellectual content. LP-C provided critical revision of the manuscript for important intellectual content. JC provided critical input in data analysis and interpretation, provided critical revision of the manuscript for important intellectual content, and supervised the study. EF is the guarantor, had full access to the data in the study, and takes responsibility for the integrity of the data and the accuracy of the data analysis. All authors read and approved the final manuscript.

## Disclaimer

The findings and conclusions in this article are those of the authors and do not necessarily represent the official position of the Centers for Disease Control and Prevention.

## Supplementary Material

Additional file 1 **Table S1.** Age-adjusted means and percentages (standard error) of sociodemographic and cardiovascular factors among 6816 U.S. adults aged 20-79 years, by categories of respiratory impairment based on postbronchodilator data, National Health and Nutrition Examination Survey 2007-2010. **Table S2**. Adjusted* prevalence ratios (95% confidence interval) for cardiovascular risk factors among 6816 U.S. adults aged 20-79 years, by categories of respiratory impairment based on postbronchodilator data, National Health and Nutrition Examination Survey 2007-2010. **Table S3**. Adjusted prevalence ratios (95% confidence interval) for 10-year cardiovascular risk >20% among U.S. adults, by categories of respiratory impairment based on postbronchodilator data, National Health and Nutrition Examination Survey 2007-2010. Click here for file
