# Supplementary material for: Elevated cardiovascular risk among adults with obstructive and restrictive airway functioning in the United States: a cross-sectional study of the National Health and Nutrition Examination Survey from 2007–2010
Source: Respir Res. 2012 Dec 13;13(1):115. doi: 10.1186/1465-9921-13-115 (PMC3546884; doi:10.1186/1465-9921-13-115)
Supplement: Additional file 1 — Table S1. Age-adjusted means and percentages (standard error) of sociodemographic and cardiovascular factors among 6816 U.S. adults aged 20-79 years, by categories of respiratory impairment based on postbronchodilator data, National Health and Nutrition Examination Survey 2007-2010. Table S2. Adjusted* prevalence ratios (95% confidence interval) for cardiovascular risk factors among 6816 U.S. adults aged 20-79 years, by categories of respiratory impairment based on postbronchodilator data, National Health and Nutrition Examination Survey 2007-2010. Table S3. Adjusted prevalence ratios (95% confidence interval) for 10-year cardiovascular risk >20% among U.S. adults, by categories of respiratory impairment based on postbronchodilator data, National Health and Nutrition Examination Survey 2007-2010. [file 1465-9921-13-115-S1.doc]

| **Table S1 Age-adjusted means and percentages (standard error) of sociodemographic and cardiovascular factors among 6816 U.S. adults aged 20-79 years, by categories of respiratory impairment based on postbronchodilator data, National Health and Nutrition Examination Survey 2007-2010** | | | | | | | |
| --- | --- | --- | --- | --- | --- | --- | --- |
|  | **Respiratory impairment** | | | |  |  |  |
|  |  |  | **Obstructive impairment** | |  |  |  |
|  | **None (N=6006)** | **Restrictive (N=493)** | **Mild (N=207)** | **Moderate/severe/very severe (N=110)** | **P restrictive‡** | **P mild OI‡** | **P moderate/severe OI‡** |
| Age (years) | 42.8 (0.3) | 50.3 (0.9) | 56.3 (1.1) | 57.9 (1.1) | 0.000 | 0.000 | 0.000 |
|  |  |  |  |  |  |  |  |
| Men (%) | 47.1 (0.6) | 56.8 (3.0) | 76.4 (4.2) | 70.2 (3.4) | 0.002 | 0.000 | 0.000 |
|  |  |  |  |  |  |  |  |
| Race or ethnicity (%) |  |  |  |  |  |  |  |
| White | 70.9 (2.2) | 55.1 (5.2) | 89.0 (3.1) | 84.1 (6.0) | 0.001 | 0.000 | 0.024 |
| African American | 9.7 (1.0) | 9.3 (1.4) | 6.8 (2.8) | 8.9 (5.2) |  |  |  |
| Mexican American | 9.1 (1.3) | 8.8 (1.9) | —§ | —§ |  |  |  |
| Other | 10.3 (1.1) | 26.8 (4.1) | —§ | —§ |  |  |  |
|  |  |  |  |  |  |  |  |
| Education (%) |  |  |  |  |  |  |  |
| <High school | 16.2 (0.9) | 19.8 (2.0) | 17.9 (4.6) | 36.5 (10.9) |  |  |  |
| High school graduate or equivalent | 22.4 (0.9) | 24.7 (3.7) | 31.2 (5.3) | 22.3 (5.8) |  |  |  |
| >High school (%) | 61.4 (1.4) | 55.5 (4.2) | 50.9 (8.3) | 41.3 (11.4) | 0.126 | 0.193 | 0.073 |
|  |  |  |  |  |  |  |  |
| Smoking status (%) |  |  |  |  |  |  |  |
| Current | 19.4 (0.7) | 24.6 (3.4) | 36.6 (4.5) | 55.8 (11.9) | 0.100 | 0.001 | 0.004 |
| Former | 23.0 (0.9) | 23.6 (2.8) | 31.7 (6.2) | 24.0 (8.5) |  |  |  |
| Never | 57.7 (1.3) | 51.8 (4.3) | 31.7 (4.5) | 20.2 (6.5) |  |  |  |
|  |  |  |  |  |  |  |  |
| Cotinine >10 ng/ml (%) | 23.4 (0.8) | 29.7 (3.7) | 47.0 (7.0) | 63.3 (11.0) | 0.068 | 0.002 | 0.001 |
|  |  |  |  |  |  |  |  |
| Hypertension (%) | 24.9 (0.9) | 34.1 (2.9) | 25.8 (3.4) | 31.2 (11.1) | 0.002 | 0.824 | 0.585 |
|  |  |  |  |  |  |  |  |
| Total cholesterol ≥5.17 mmol/L (%) | 44.8 (0.9) | 41.1 (2.5) | 48.4 (4.9) | 46.6 (10.0) | 0.205 | 0.486 | 0.867 |
|  |  |  |  |  |  |  |  |
| High-density lipoprotein cholesterol <1.03 mmol/L in men, <1.29 mmol/L in women | 31.8 (1.1) | 49.7 (3.0) | 22.5 (5.4) | 35.4 (11.5) | 0.000 | 0.098 | 0.750 |
|  |  |  |  |  |  |  |  |
| Non-high-density lipoprotein cholesterol ≥130 mg/dl | 61.6 (0.7) | 61.6 (2.9) | 67.4 (3.8) | 62.4 (10.0) | 0.992 | 0.139 | 0.940 |
|  |  |  |  |  |  |  |  |
| Low-density lipoprotein cholesterol ≥2.59 mmol/L* | 67.6 (1.0) | 67.9 (4.1) | 81.9 (3.4) | 60.7 (11.9) | 0.959 | 0.000 | 0.557 |
|  |  |  |  |  |  |  |  |
| C-reactive protein >3 mg/l | 29.9 (0.7) | 48.6 (3.2) | 18.2 (3.4) | 36.5 (8.5) | 0.000 | 0.002 | 0.432 |
|  |  |  |  |  |  |  |  |
| FPG ≥7.0 mmol/L or diagnosed diabetes† | 8.9 (0.7) | 25.6 (3.8) | 2.7 (0.9) | 7.1 (2.6) | 0.000 | 0.000 | 0.499 |
|  |  |  |  |  |  |  |  |
| Diagnosed diabetes (%) | 6.0 (0.4) | 18.5 (2.3) | 3.7 (2.5) | 5.4 (1.6) | 0.000 | 0.418 | 0.685 |
|  |  |  |  |  |  |  |  |
| Body mass index ≥30 kg/m2 | 34.6 (1.0) | 51.0 (2.9) | 19.9 (3.9) | 24.4 (8.9) | 0.000 | 0.001 | 0.257 |
|  |  |  |  |  |  |  |  |
| Abdominal obesity (%) | 52.8 (1.0) | 64.8 (2.8) | 41.2 (4.4) | 43.2 (9.3) | 0.000 | 0.012 | 0.296 |
|  |  |  |  |  |  |  |  |
| Leisure-time physical activity <150 min/week | 59.7 (1.4) | 70.1 (2.8) | 56.0 (7.2) | 48.4 (4.0) | 0.002 | 0.593 | 0.006 |
|  |  |  |  |  |  |  |  |
| Congestive heart failure (%) | 0.8 (0.1) | 3.3 (0.8) | —§ | —§ | 0.004 | 0.399 | 0.795 |
|  |  |  |  |  |  |  |  |
| 10-Year cardiovascular risk† |  |  |  |  |  |  |  |
| <10% | 77.3 (0.9) | 69.2 (3.8) | 73.1 (3.5) | 73.9 (4.6) |  |  |  |
| 10-20% | 14.6 (0.9) | 16.2 (2.7) | 13.3 (2.9) | 12.7 (4.7) |  |  |  |
| >20% | 8.1 (0.5) | 14.6 (2.2) | 13.6 (2.4) | 13.4 (3.1) | 0.003 | 0.023 | 0.124 |
|  |  |  |  |  |  |  |  |
| *Fasting subsample. Sample sizes are 2633, 229, 105, and 44, respectively. | | | | | | | |
| †Fasting subsample. Sample sizes are 2679, 236, 105, and 45, respectively. | | | | | | | |
| OI = obstructive impairment. | | | | | | | |
| ‡P-values are for comparison with participants with no respiratory impairment. | | | | | | | |
| §Relative standard error >30%. | | | | | | | |

| **Table S2 Adjusted* prevalence ratios (95% confidence interval) for cardiovascular risk factors among 6816 U.S. adults aged 20-79 years, by categories of respiratory impairment based on postbronchodilator data, National Health and Nutrition Examination Survey 2007-2010** | | | | | |
| --- | --- | --- | --- | --- | --- |
|  | **Respiratory impairment** | | | |  |
|  |  |  | **Obstructive impairment** | |  |
|  | **None** | **Restrictive** | **Mild** | **Moderate/severe/very severe** | **P†** |
| Current smoking | 1.00 | 1.22 (0.99, 1.50) | 1.67 (1.33, 2.09) | 2.41 (1.89, 3.07) | <0.001 |
|  |  |  |  |  |  |
| Cotinine >10 ng/ml | 1.00 | 1.22 (1.01, 1.46) | 1.60 (1.31, 1.95) | 2.23 (1.80, 2.76) | <0.001 |
|  |  |  |  |  |  |
| Hypertension | 1.00 | 0.98 (0.86, 1.13) | 0.99 (0.76, 1.29) | 0.85 (0.62, 1.16) | 0.769 |
|  |  |  |  |  |  |
| Total cholesterol ≥5.17 mmol/L | 1.00 | 0.89 (0.76, 1.04) | 0.85 (0.71, 1.01) | 0.93 (0.72, 1.20) | 0.153 |
|  |  |  |  |  |  |
| High-density lipoprotein cholesterol <40 mg in men, <1.29 mmol/L in women | 1.00 | 1.17 (1.02, 1.33) | 0.89 (0.65, 1.21) | 1.02 (0.75, 1.38) | 0.163 |
|  |  |  |  |  |  |
| Non-high-density lipoprotein cholesterol ≥130 mg/dl | 1.00 | 0.91 (0.82, 1.00) | 0.85 (0.75, 0.97) | 0.97 (0.81, 1.16) | 0.038 |
|  |  |  |  |  |  |
| Low-density lipoprotein cholesterol ≥2.59 mmol/L‡ | 1.00 | 1.68 (1.21, 2.32) | 0.36 (0.18, 0.72) | 0.74 (0.37, 1.47) | 0.798 |
|  |  |  |  |  |  |
| C-reactive protein >3mg/l | 1.00 | 1.22 (1.05, 1.41) | 0.72 (0.54, 0.96) | 1.49 (1.17, 1.88) | 0.001 |
|  |  |  |  |  |  |
| Diagnosed diabetes | 1.00 | 1.85 (1.41, 2.42) | 0.43 (0.20, 0.92) | 1.02 (0.57, 1.82) | <0.001 |
|  |  |  |  |  |  |
| Diagnosed diabetes of fasting plasma glucose ≥7.0 mmol/L§ | 1.00 | 0.93 (0.81, 1.05) | 0.98 (0.85, 1.14) | 0.99 (0.80, 1.23) | <0.001 |
|  |  |  |  |  |  |
| Body mass index ≥30 kg/m^2^ | 1.00 | 1.18 (1.06, 1.31) | 0.62 (0.44, 0.87) | 0.68 (0.41, 1.14) | 0.005 |
|  |  |  |  |  |  |
| Abdominal obesity | 1.00 | 1.08 (1.01, 1.17) | 0.94 (0.81, 1.09) | 0.86 (0.71, 1.05) | 0.082 |
|  |  |  |  |  |  |
| Leisure-time physical activity <150 min/week | 1.00 | 1.06 (0.98, 1.14) | 1.06 (0.94, 1.20) | 0.99 (0.84, 1.16) | 0.412 |
|  |  |  |  |  |  |
| *Adjusted for age, gender, race or ethnicity, educational status, smoking status (except for cotinine concentration), cotinine concentration (except smoking status), systolic blood pressure (except hypertension), high-density lipoprotein cholesterol concentration (except non-high-density lipoprotein cholesterol concentration, non-high-density lipoprotein cholesterol concentration (except high-density lipoprotein cholesterol, C-reactive protein concentration, diagnosed diabetes, body mass index (except waist circumference), waist circumference (except body mass index), leisure-time physical activity, and history of congestive heart failure. Hypercholesterolemia was adjusted for all above factors except lipids. Low-density lipoprotein cholesterol was adjusted for all factors except non-high-density lipoprotein cholesterol. | | | | | |
| †P-value for Satterthwaite adjusted F-test. | | | | | |
| ‡Sample size is 3011. | | | | | |
| §Sample size is 3065. | | | | | |

| **Table S3 Adjusted prevalence ratios (95% confidence interval) for 10-year cardiovascular risk >20% among U.S. adults, by categories of respiratory impairment based on postbronchodilator data, National Health and Nutrition Examination Survey 2007-2010** | | | | |
| --- | --- | --- | --- | --- |
|  | **Respiratory impairment** | | | |
|  |  |  | **Obstructive impairment (OI)** | |
|  | **None** | **Restrictive** | **Mild** | **Moderate/severe/**  **very severe** |
|  |  |  |  |  |
| **Age 50-74 years** |  |  |  |  |
| No. risk >20% / no. at risk* | 235 / 950 | 56 / 131 | 29 / 66 | 14 / 33 |
| Median age (years) | 57 | 60 | 59 | 59 |
| Unadjusted prevalence (standard error) (%) | 16.9 (1.2) | 34.8 (5.6) | 27.5 (7.8) | 30.1 (8.4) |
| Unadjusted prevalence ratio | 1.00 | 2.05 (1.58, 2.66) | 1.62 (0.91, 2.88) | 1.77 (0.97, 3.26) |
| Adjusted prevalence ratio† | 1.00 | 1.64 (1.20, 2.24) | 1.77 (0.99, 3.15) | 1.82 (0.98, 3.36) |
|  |  |  |  |  |
| **Age 55-74 years** |  |  |  |  |
| No. risk >20% / no. at risk* | 208 / 677 | 51 / 105 | 27 / 58 | 10 / 23 |
| Median age (years) | 61 | 62 | 61 | 62 |
| Unadjusted prevalence (standard error) (%) | 23.3 (1.6) | 41.6 (6.6) | 31.5 (8.8) | 33.6 (9.2) |
| Unadjusted prevalence ratio | 1.00 | 1.79 (1.37, 2.33) | 1.35 (0.79, 2.32) | 1.44 (0.78, 2.68) |
| Adjusted prevalence ratio† | 1.00 | 1.37 (0.98, 1.90) | 1.55 (0.92, 2.61) | 1.64 (0.93, 2.89) |
|  |  |  |  |  |
| *Unweighted numbers. |  |  |  |  |
| †Adjusted for race or ethnicity, education, body mass index, C-reactive protein, and leisure-time physical activity. | | | | |
